# The Targets of Deep Brain Stimulation in the Treatment of Treatment‐Resistant Depression: A Review

**DOI:** 10.1002/brb3.70505

**Published:** 2025-05-05

**Authors:** Jianyang Dong, Mengying Dai, Zinan Guo, Ting Xu, Fangming Li, Jianjun Li

**Affiliations:** ^1^ Department of Rehabilitation Shenzhen University, Shenzhen University General Hospital Shenzhen China; ^2^ Department of Rehabilitation Shenzhen Children's Hospital Shenzhen China; ^3^ Department of Neurology, Guangzhou First People's Hospital South China University of Technology Guangzhou China; ^4^ Department of Neurology Shenzhen University, Shenzhen University General Hospital Shenzhen China

**Keywords:** deep brain stimulation, AMPAR, neuromodulation, treatment‐resistant depression, reward pathway

## Abstract

**Purpose:**

The purpose of this review is to evaluate the current state and potential future directions of deep brain stimulation (DBS) therapy for treatment‐resistant depression (TRD), a condition that significantly impacts patients' quality of life and for which conventional treatments are often ineffective.

**Method:**

This review synthesizes evidence from clinical trials and preclinical studies published in five years, identified through PubMed searches using keywords (“Deep Brain Stimulation” OR DBS) AND (“Treatment‐Resistant Depression” OR TRD). Included studies encompassed clinical research (randomized/non‐randomized trials, cohort studies) and mechanistic preclinical studies, excluding non‐English publications and nonhuman experiments. Screening prioritized neuroanatomical targets (e.g., SCG, NAcc) and stimulation parameter optimization data. Examining the therapeutic mechanisms of DBS, the neuroanatomical targets utilized, and the clinical outcomes observed. It also discusses the challenges faced in DBS application and proposes potential technological advancements, such as closed‐loop therapy and fiber tracking technology.

**Finding:**

Preliminary evidence exists regarding the efficacy and safety of DBS in the treatment of TRD in the subcortical cingulate gyrus (SCG), nucleus accumbens (NAcc), ventral capsule/ventral striatum (VC/VS), anterior limb of the internal capsule (ALIC), and so forth. Nevertheless, the optimal stimulation target remains undetermined. The review highlights the complexity of TRD and the need for personalized treatment strategies, noting that genetic, epigenetic, and neurophysiological changes are implicated in DBS's therapeutic effects.

**Conclusion:**

In conclusion, while DBS for TRD remains an experimental therapy, it offers a unique and potentially effective treatment option for patients unresponsive to traditional treatments. The review emphasizes the need for further research to refine DBS targets and parameters, improve patient selection, and develop personalized treatment plans to enhance efficacy and safety in TRD management.

## Graphical abstract

Schematic Representation of the DBS Procedure for the Management of TRD. The DBS electrode is electrically stimulated by a long lead. Potential mechanisms of action are presented below. The right side highlights the key summary of the article.

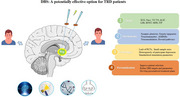

## 1 Introduction

Depression is the most prevalent mental disorder, with patients often experiencing aversion, despair, hallucinations, and suicidal attempts and behaviors, posing a serious threat to their health. The incidence rate can reach 15% to 20%, with a recurrence rate as high as 30% to 40% (Narang et al. 2016). According to estimates from the WHO, depression is projected to emerge as the primary cause of disease burden globally by 2030. Among them, treatment‐resistant depression (TRD) is defined as depression that does not show significant improvement after at least two different mechanisms of antidepressants have been administered in sufficient amounts and for a standardized treatment course (Gaynes et al. 2020). Even among individuals receiving standard care for depression, approximately 30% develop TRD (Papp et al. 2022), which not only increases the cost of treatment but also imposes a heavier burden on the patient (Johnston et al. 2019; Shin et al. 2020).

In recent years, there has been a growing recognition of the limitations of drug and cognitive therapy in the treatment of TRD. As a result, there is an increasing interest in exploring nondrug therapies such as transcranial magnetic stimulation (TMS), vagus nerve electrical stimulation (VNS), electroconvulsive therapy, epidural cortical stimulation (ECS), and other alternative treatments (Raymaekers et al. 2017). This shift towards nondrug therapies reflects a need for more effective options for individuals with TRD.

Unfortunately, despite growing clinical interest in non‐pharmacological therapies such as VNS and TMS due to the limited efficacy of standard drug and cognitive therapies for TRD, there is currently no definitive recommendation for these treatments (Johnson and Wilson 2018; Lefaucheur et al. 2020). Among them, deep brain stimulation (DBS) is the most promising but invasive treatment for TRD patients and may be the last choice for patients who have failed other less invasive treatments (Mayberg et al. 2005). Deep brain electrodes are implanted into specific neural targets using a stereotactic frame in order to treat depression by stimulating the imbalanced excitation‐inhibition neural circuit (Chiken and Nambu 2016). Meta‐analysis and review show that approximately 50% of TRD patients are effectively treated with DBS, as measured by clinician‐rated scales such as the Montgomery–Åsberg Depression Rating Scale (MADRS) and Hamilton Depression Rating Scale (HAM‐D), which serve as primary outcome measures in most clinical trials. However, the reliance on clinician assessments and partially self‐reported components in these scales may introduce subjectivity or reporting biases (Dandekar et al. 2018; Sadeghzadeh et al. 2024).

At present, multiple DBS targets have entered clinical trials, such as the subcortical cingulate gyrus (SCG), nucleus accumbens (NAcc), anterior limb of the internal capsule (ALIC), bed of the striatum (BNST), inferior thalamus (ITP), lateral habenular nucleus (LHb), medial forebrain tract (MFB), and so forth. Despite this, the specific mechanism and optimal target of DBS in the treatment of TRD are still unclear. Here, we provide updated knowledge substantiating the suitability of the potential mechanism and each of the current DBS targets for treating depression. Finally, current challenges and potential development directions were proposed, including closed‐loop therapy and combined fiber tracking technology. This review can provide a unique and valuable contribution to the application of DBS in TRD neurological diseases.

## 2 Potential Therapeutic Mechanism of DBS in TRD Treatment

### 2.1 Changes in Neuronal Soma and Synapses

Early studies on DBS have shown that voltage‐gate blockade, membrane hyperpolarization, depletion of neurotransmitters, and release of inhibitory neurotransmitters may help to inhibit abnormal neuronal activity in the short term, partially explaining the short‐term relief of symptoms in patients with depression (Anderson et al. 2006; Aum and Tierney 2018), but this does not explain the mechanism by which months of stimulation improves depressive symptoms. Lujan et al. (2008) proposed that alterations in synaptic plasticity may underlie the sustained amelioration of depressive symptoms observed in patients during long‐term treatment. Shen et al. (2003) conducted high‐frequency stimulation on subthalamic nucleus neurons in rats and observed modifications in synaptic plasticity, including long‐term potentiation and depression, thereby providing experimental evidence for electrical stimulation‐induced changes in synaptic plasticity. Aldehri et al. (2019) conducted a 7‐week fornix DBS study on mice, and subsequent histopathological examination revealed decreased levels of synaptophysin in the hippocampal CA1 and CA3 subregions, indicating that fornix DBS can induce alterations in synaptic plasticity, characterized by long‐term inhibition. Furthermore, Pohodich et al. (2018) performed genetic analysis on mice post‐DBS and confirmed that DBS elicited the expression, transcription, and RNA splicing of numerous genes associated with neural plasticity at the genetic level. The biological pathway analysis revealed a strong correlation between the expressed proteins and key processes involved in neurogenesis, neuronal morphological changes, and synaptic function. However, previous studies only confirmed the impact of electrical stimulation on synaptic plasticity changes without elucidating the underlying mechanism linking these changes to the relief of depressive symptoms.

### 2.2 Genetic and Epigenetic Changes

Pohodich et al. (2018) conducted a comparative analysis between the downregulated gene database extracted from postmortem brain tissue of TRD patients and the upregulated gene database in mice after DBS, revealing a 17% overlap rate among 325 genes. Furthermore, they also compared the gene expression profiles of mice after DBS with those following treatment with fluoxetine and physical exercise, demonstrating significant overlap in gene expression patterns. These findings suggest that the transcriptional program activated by DBS partially overlaps with that induced by fluoxetine and exercise. This study represents the pioneering investigation into the genetic underpinnings of DBS for TRD, elucidating a shared genetic pathway with certain pharmacological and exercise interventions. Currently, limited research exists on the genetic mechanisms underlying DBS for refractory depression, and findings in this area may serve as robust experimental evidence for future DBS‐based treatments of TRD.

Furthermore, Papp et al. (2019) conducted a genetic analysis on depression model rats subjected to DBS, including Wistar and Wistar Kyoto (WKY) rats, with WKY rats being regarded as an antidepressant‐treated rat model akin to patients suffering from TRD (Lahmame et al. 1997). The depression symptoms of both groups improved following DBS, and DBS reversed the downregulation of *Egr1*, *Htr7*, and *MMP‐9* genes in the ventral hippocampus of Wistar rats. However, no change was observed in the expression levels of related downregulated genes in WKY mice, suggesting a potential specific gene pathway for DBS treatment of TRD. Further experiments should include additional candidate genes to further elucidate drug resistance mechanisms in TRD and subsequently enhance drug therapy and DBS treatment.

### 2.3 Activation of Prefrontal α‐Amino‐3‐Hydroxy‐5‐Methyl‐4‐Isoxazole‐Propionic Acid Receptors and Release of Neuromodulator

Jiménez‐Sánchez et al. (2016) observed the release levels of activated brain regions and neuromodulators in mice following DBS, revealing that glutamate efflux from the lower prefrontal lobe margin induced activation of prefrontal α‐amino‐3‐hydroxy‐5‐methyl‐4‐isoxazole‐propionic acid receptor (AMPAR) activation, thereby stimulating the prefrontal lobe's influence on the brainstem and subsequently elevating serotonin, dopamine, and norepinephrine levels in the prefrontal cortex (PFC) to achieve an antidepressant effect. The experiment demonstrates that the activation of AMPAR receptors is both necessary and sufficient for DBS to exert its antidepressant effect. This brain region currently represents one of the commonly targeted areas for DBS treatment of TRD. AMPARs could potentially serve as a biomarker for assessing the efficacy of DBS treatment, although this still requires validation through relevant clinical trials.

### 2.4 Subcortical and Cortical Reward Pathways

The medial forebrain bundle (MFB) and ventral tegmental area (VTA) are pivotal subcortical structures within the human reward system, exerting a significant influence on both emotional disorders, such as depression and obsessive–compulsive disorder. Coenen et al. (2018) postulated that the stimulation of the superior lateral medial forebrain bundle (slMFB) could potentially activate both ascending and descending fibers entering and exiting the VTA, as well as their corresponding projection areas, thereby potentially ameliorating depressive symptoms. Although small‐sample clinical trials suggest the efficacy of slMFB‐DBS, further microanatomical evidence is imperative to substantiate its relevance (Coenen, Sajonz, et al. 2018).

### 2.5 Changes in Neuroelectrophysiological

In 2013, Ewing and Grace (2013) postulated that the alleviation of psychiatric symptoms through DBS may be partially ascribed to the augmentation of neuroelectrophysiological synchrony in interconnected brain regions. Jia et al. (2019) employed electrical stimulation of the ventral PFC in a depression model of rats, and preoperative local field potential (LFP) detection revealed that the baseline power of β and γ waves was lower in mice compared to the normal control group. Following the procedure, there was a significant enhancement in γ and β wave oscillations within the ventral PFC and hippocampus of rats, accompanied by an observed increase in synchronization strength. Accordingly, it is postulated that the augmentation of β–γ wave synchrony within the relevant cerebral regions may constitute one of the underlying mechanisms of DBS in depression treatment.

### 2.6 Changes in Adrenocorticotropic Hormone, Nerve Growth Factor, and Cytokine Levels

The hyperactivity of the hypothalamic–pituitary–adrenal (HPA) axis, reduced levels of neurotrophic factors, and elevated levels of inflammatory factors are all potential contributors to depressive symptoms and are classified as possible etiologies for depressive disorders. Dandekar et al. (2019) conducted a 7‐day stimulation of the MFB in rats with depression. They compared the levels of adrenocorticotropic hormone, cranial nerve growth factor, and inflammatory factors in peripheral blood, cerebrospinal fluid, and hippocampus after modeling and after operation. The findings demonstrated that DBS could reverse the downregulation of neurotrophic factors, upregulate adrenocorticotropic hormone levels, and mitigate overexpression of inflammatory factors such as IL‐1, IL‐6, TNF‐α, and IFN‐γ in mice with depression. The application of DBS can effectively ameliorate depressive symptoms through three mechanisms: inhibition of aberrant HPA axis activity, downregulation of inflammatory factor expression, and promotion of brain neurotrophic factor secretion.

## 3 Targets of DBS for TRD

**FIGURE 1:**
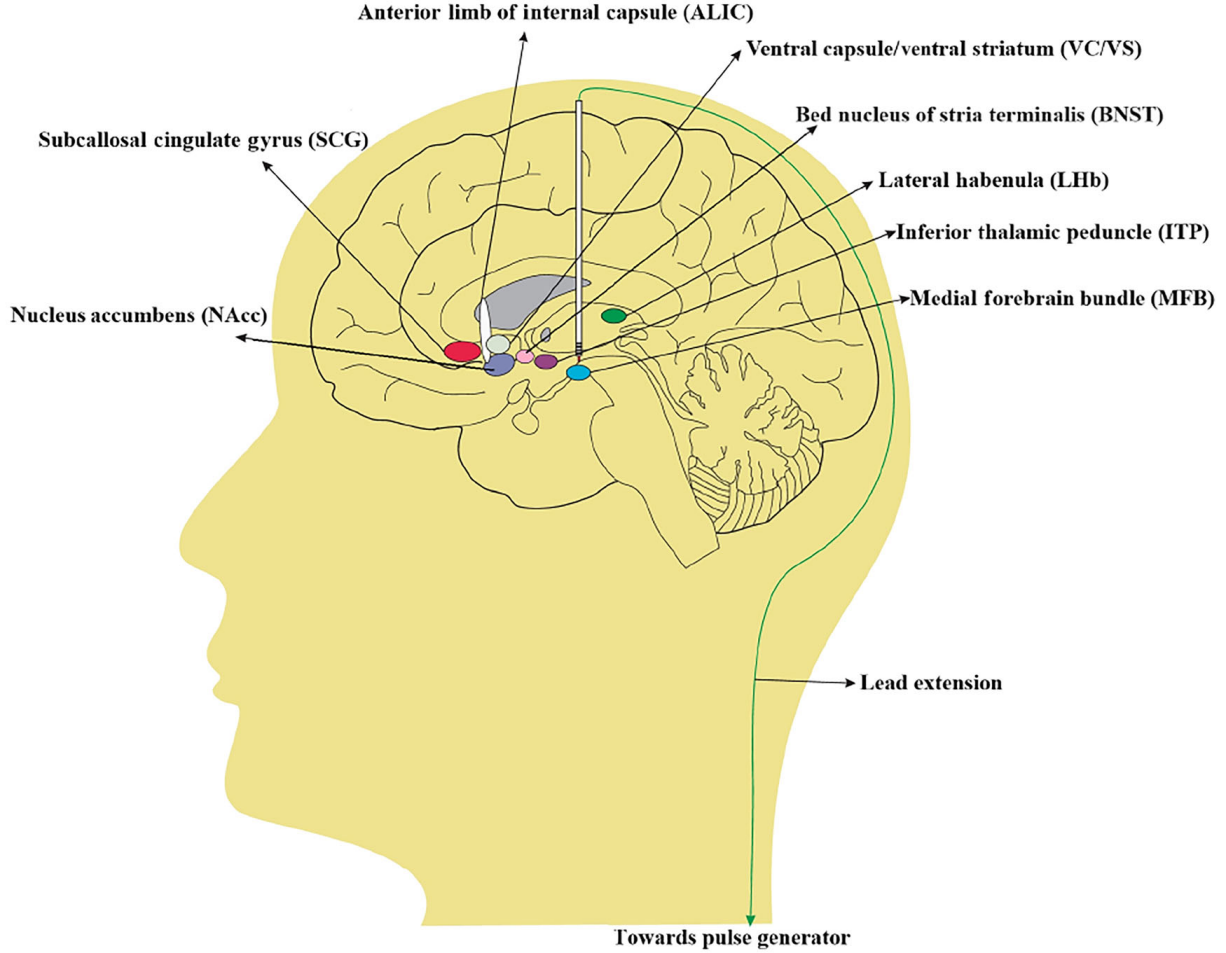
Schematic representation of eight DBS targets tested for the management of TRD. The DBS electrode is electrically stimulated by a long lead. DBS, deep brain stimulation; TRD, treatment‐resistant depression. Image adapted from Ref. (Dandekar et al. 2018). Copyright 2018 Nature.

The selection of DBS targets is often based on functional targets in the emotional regulation circuit, and the functional nuclei of the target are closely related to monoamine neuronal nuclei. In addition, the effect of improving emotions can be observed in animal models or other treatments for neuropsychiatric diseases (Drobisz and Damborská 2019). Currently, the optimal stimulation target for DBS treatment of TRD is still under investigation (Figure 1). The clinical studies of DBS for TRD in recent years are summarized in Table 1.

**TABLE 1:** Summary of DBS targets and clinical trials for the treatment of TRD.

Target	Study type	*N*	Age (mean)	Follow‐up (months)	Response rate	Ref.
VC/VS						
		18	—	24	72.2%	van der Wal et al. (2020)
	RCT	25	—	13	60%	Bergfeld et al. (2016)
	RCT	30	47.7	24	46.6%	Dougherty et al. (2015)
SCG						
	RCT	5	—	36	20%	Conroy et al. (2018)
	OLS	28	45	48–96	≥ 80%	Crowell et al. (2019)
	RCT	9	46	12	44.4%	Eitan et al. (2018)
	RCT	8	48.3	28	6 months: 50%, 12 months: 57.2%, 24 months: 66.7%	Merkl et al. (2018)
	OLS	11	48.7	12	81.8%	Riva‐Posse et al. (2018)
	RCT	90	50.5	24	12 months: 43.3%, 18 months: 61.2%, 24 months: 64.4%	Holtzheimer et al. (2017)
	OLS	20	47.4	12	55%	McInerney et al. (2017)
	OLS	5	45.2	24	20%	Accolla et al. (2016)
	RCT	5	46.35	6	80%	Puigdemont et al. (2015)
	OLS	8	—	1	1 week:100%, 4 weeks: 100%	Perez‐Caballero et al. (2014)
	CR	1	78	9	100%	Torres et al. (2013)
	OLS	6	50.7	6–9	66.7%	Merkl et al. (2013)
	OLS	4	50.25	9	75%	Ramasubbu et al. (2013)
	OLS	17	42	24	6 months: 59%, 12 months:72%, 24 months: 91.7%	Holtzheimer et al. (2012)
	OLS	8	47.4	12	62.5%	Puigdemont et al. (2012)
	OLS	12	40.4	1–6	50%	Broadway et al. (2012)
	OLS	21	47.3	12	1 month: 57%, 6 months: 48%, 12 months: 29%	Lozano et al. (2012)
	OLS	20	47.4	36–72	12 months: 81.3%, 24 months: 61.6%, 36 months: 75%, Last: 64.3%	Kennedy et al. (2011)
	CR	1	60	18	100%	Guinjoan et al. (2010)
	CR	1	64	12	100%	Puigdemont et al. (2009)
	OLS	20	—	12	55%	Hamani et al. (2009)
	CR	1	55	30	100%	Neimat et al. (2008)
	OLS	20	47.4	12	95%	Lozano et al. (2008)
	OLS	6	46	6	66.7%	Mayberg et al. (2005)
NAcc						
	OLS	6	4	4	50%	Millet et al. (2014)
	OLS	11	48.3	12–48	45.4	Bewernick et al. (2012)
	OLS	10	48.6	12	20%	Bewernick et al. (2010)
	OLS	3	46.7	5.5	100%	Schlaepfer et al. (2008)
LHb						
	OLS	7	39.0	1–12	57.2%	Zhang et al. (2022)
	CR	1	64	15	100%	Sartorius et al. (2010)
BNST						
	OLS	5	44.6	18–24	6 months: 0%, 12 months: 20%, Last: 60%	Fitzgerald et al. (2018)
	OLS	5	50.0	36–97	71.4%	Raymaekers et al. (2017)
	CR	1	36	12	/	Cassimjee et al. (2018)
	CR	1	36	12	/	Blomstedt et al. (2017)
MFB						
	OLS	2	58.5	6	0%	Davidson et al. (2020)
	OLS	6	50.2	13	80%	Fenoy et al. (2018)
	OLS	8	41.9	3–12	75%	Bewernick et al. (2017)
	CR	1	60	24	100%	Blomstedt et al. (2017)
	OLS	4	46.3	6.5	66.7%	Fenoy et al. (2016)
	OLS	7	42.6	3–8	85.7%	Schlaepfer et al. (2013)
ITP						
	OLS	7	50	92	57%	Raymaekers et al. (2017)
	CR	1	—	36	100%	Jiménez et al. (2013)
	CR	1	—	18	100%	Jiménez et al. (2007)
	CR	1	49	24	100%	Jiménez et al. (2005)
ALIC						
	OLS	25	53.1	13	60% (15/25)	Bergfeld et al. (2016)
	OLS	30	47.7	12–24	12 months: 33%, 24 months: 43.3%	Dougherty et al. (2015)
	CR	1	43	48	100%	Strong et al. (2012)
	OLS	17	46.3	14–67	3 months: 88%, 6 months: 6%, 12 months: 94%, Last: 70%	Malone 2010)
	OLS	15	46.3	12	93.3%	Malone et al. (2009)

Abbreviations: CR, case report; OLS, open‐label trial, RCT, randomized controlled trial.

### 3.1 Subcallosal Gyrus

The SCG, or Broadman 25 (BA25), is a cerebral gyrus located between the inferior cingulate sulcus of the corpus callosum and the sulcus of the corpus callosum. SCG exerts regulatory control over corresponding brain regions through direct or indirect neural pathways, thereby participating in modulating the reward–feedback loop in patients with depression (Hamani et al. 2011). Previous studies have demonstrated a correlation between depression and heightened metabolic activity in SCG, as well as dysfunction within the cortical limbic network (Mayberg et al. 1999). Furthermore, SCG‐DBS has shown potential in ameliorating depressive symptoms, particularly anhedonia, suggesting its preferential utility for TRD patients with prominent reward‐processing deficits (Eggers 2014). This aligns with evidence that SCG connectivity to the ventral striatum and PFC supports its role in addressing motivational impairments (Riva‐Posse et al. 2014). Simultaneously, studies have demonstrated that SCG‐DBS can effectively suppress γ oscillation, enhance θ–γ coupling, and facilitate the release of γ‐aminobutyric acid neurotransmitter, thereby exerting a regulatory impact (Sun et al. 2015). Consequently, SCG‐DBS stimulation may play a pivotal role in normalizing brain network activity rhythm associated with the neurobiology of depression (Dandekar et al. 2018).

In 2005, Mayberg et al. (2005) initially proposed the utilization of SCG as a treatment for TRD. Out of the six patients with TRD included in their study, remission was achieved by four patients after a span of 6 months during the open trial, indicating promising therapeutic prospects (Figee et al. 2022). Subsequently, in 2008, they conducted further follow‐up on these initial 6 patients along with an additional cohort of 14 patients. The outcomes revealed that treatment response was observed in 60% of the patients, complete remission was attained by 35% of them, and this effect could be sustained for over a period exceeding 12 months without any notable side effects or intolerance (Lozano et al. 2008). In 2011, the same research group continued to report on the long‐term efficacy of SCG‐DBS. The response rates of patients with TRD were found to be 62.5%, 46.2%, and 75% at an average follow‐up duration of 1, 2, and 3 years, respectively. Furthermore, at the last follow‐up conducted between 3 and 6 years post‐SCG‐DBS, TRD patients exhibited an average response rate of 64.3% (Mohr et al. 2011). Subsequently, other research groups initiated clinical trials of SCG‐DBS for TRD. For instance, Puigdemont et al. (2012) conducted clinical trials on eight TRD patients and reported complete symptom relief in four patients after 1 year. Further randomized, double‐blind, controlled clinical trials confirmed that four out of five TRD patients achieved complete symptom relief (Puigdemont et al. 2015), with high‐frequency electrical stimulation demonstrating superior long‐term antidepressant effects compared to low‐frequency electrical stimulation (Hamani et al. 2011). In terms of long‐term efficacy, Crowell et al. (2019) conducted a follow‐up study for up to 8 years, revealing that the effective rate of SCG‐DBS treatment remained consistently above 50%, with a complete remission rate exceeding 30% since the second year. These findings demonstrate the safety and effectiveness of SCG‐DBS as a therapeutic approach for TRD. However, a recent randomized, double‐blind study of 90 patients revealed no statistically significant difference in the response rate (20%) to SCG‐DBS stimulation during the double‐blind control phase compared with the control group (17%) (Holtzheimer et al. 2017). The same conclusion was corroborated by another double‐blind trial involving eight patients with TRD (Merkl et al. 2018). This discrepancy between short‐term and long‐term outcomes may arise from factors such as insufficient time for stimulation parameter optimization, variability in the anatomical precision of lead placement, and individual neurocircuit heterogeneity. However, considering long‐term efficacy, treatment response rates at 12, 18, and 24 months after DBS stimulation were observed in 29%, 53%, and 49% of patients, respectively, underscoring that sustained clinical benefits often emerge beyond the acute phase (Holtzheimer et al. 2017). In conclusion, the SCG exhibits extensive connectivity with regions implicated in emotional and motivational processing, encompassing pathways to the NAcc, thalamus, and limbic structures, thereby underscoring its pivotal role in emotion regulation. Furthermore, the SCG is interconnected with other DBS targets, including the ventral capsule/ventral striatum (VC/VS) and the MFB (Riva‐Posse et al. 2014).

### 3.2 Nucleus Accumbens

The NAcc is located external to the inferior septum, representing an extension of the caudate nucleus towards the inner and lower regions. It plays a pivotal role as a crucial basal forebrain nucleus involved in regulating emotional responses (Park et al. 2019). The electrical stimulation of the NAcc has been shown to normalize hypermetabolism in the PFC, including the SCG and orbitofrontal cortex, thereby regulating depression (Bewernick et al. 2010). This finding aligns with the observed effects of SCG‐DBS in reducing abnormal cortical hypermetabolism (Lozano et al. 2008). Therefore, it is hypothesized that the antidepressant effect of SCG‐DBS may potentially mediate the antidepressant effect of NAcc concurrently (Bewernick et al. 2010). Currently, the efficacy of NAcc‐DBS in treating TRD has been substantiated by multiple clinical trials. Schlaepfer et al. (2008) initially reported the short‐term impact of NAcc‐DBS on three TRD patients, demonstrating that when the stimulator was activated, there was an improvement in their clinical scores; conversely, when the stimulator was deactivated, their clinical scores deteriorated. Subsequently, (Bewernick et al. 2012) reported on a cohort of 10 TRD patients who underwent NAcc‐DBS; following 12 months of electrical stimulation, the response rate and remission rate among TRD patients were determined to be 45% and 9%, respectively. Additionally, they observed a decrease in brain metabolism within the regions encompassing the SCG, amygdala, and prefrontal lobe (Bewernick et al. 2010). Meanwhile, five patients (45.6%) demonstrated sustained improvement without deterioration after a 4‐year follow‐up period (Bewernick et al. 2012). In the open trial of NAcc‐DBS conducted by Millet et al. (2014), significant improvement was observed in three out of six patients following NAcc‐DBS stimulation. Positron emission tomography‐computed tomography (PETCT) revealed decreased glucose metabolism in the posterior cingulate cortex, left superior frontal gyrus, middle frontal gyrus, and bilateral cerebellum, accompanied by increased glucose metabolism in the right anterior cingulate cortex, bilateral superior frontal gyrus, and left medial prefrontal lobe (Millet et al. 2014). No evidence of cognitive decline was found 1 year after NAcc‐DBS stimulation, indicating its favorable safety profile (Grubert et al. 2011). These newly emerging findings suggest that the NAcc is functionally impaired in depression, especially in the reward circuit, which highlights its potential as a DBS target for treating symptoms such as anhedonia and motivational deficits in depression. Clinical trials report NAcc‐DBS efficacy in patients with severe anhedonia but limited response in those with primary psychomotor retardation (Bewernick et al. 2010).

### 3.3 Ventral Capsule/Ventral Striatum

VC/VS is closely connected to the NAcc, including various white matter tracts and subcortical gray matter structures related to emotional behavior. As early as 2008, a multicenter open trial treated 15 TRD patients with DBS targeting VC/VS (Malone et al. 2009). After 6 months to 4 years of follow‐up, the results showed that six cases (40%) were effective at 6 months, and eight cases (53%) were effective at 48 months. This provides hope for DBS targeting VC/VS to treat TRD. A recent study has reported that 25 patients with TRD underwent DBS targeting the VC and were followed up for a period of 2 years (van der Wal et al. 2020). The efficacy of the treatment was assessed using the HAM‐D, MADRS, and a self‐rating scale for depressive symptoms. The results indicated that 11 patients (44.4%) experienced significant improvement, and this effect remained stable over time. However, Dougherty et al. (2015) conducted a true–false stimulation control test on 34 TRD patients (true stimulation group: 18 cases, sham stimulation group: 16 cases) with VC/VS as the target for 16 weeks of DBS treatment, and the study showed no statistically significant difference in efficacy between the two groups, which may be related to the shorter treatment time. In addition, a case report of a TRD patient developed Tourette‐like symptoms associated with stimulation voltage after 32 months of DBS treatment with VC/VS as the target, which was relieved by adjusting the stimulation voltage scheme (Camprodon et al. 2020).

### 3.4 Anterior Limb of the Internal Capsule

The ALIC is a pivotal component within the cortical–striatal–thalamocortical (CSTC) circuit, exerting regulatory effects on various structures, including the orbitofrontal gyrus (Van Laere et al. 2006), inferior cingulate gyrus of the corpus callosum, basal ganglia, and other regions that are critically involved in reward processing and motivational processes. The ALIC lies adjacent to the NAcc, a core constituent of the reward circuit, and the bed nucleus of the stria terminalis (BNST), which is implicated in anxiety and stress responses. The association of ALIC with the reward circuit accentuates its significance in anhedonia, a cardinal symptom of depression (Nanda et al. 2017; Figee et al. 2013). The ALIC was initially developed for the treatment of obsessive–compulsive disorder; however, these studies have also revealed its significant efficacy in alleviating depressive symptoms. Consequently, these findings prompted the researchers to initiate a pilot clinical trial investigating the potential of ALIC‐DBS. The findings demonstrated that the response rate and complete remission rate among patients with TRD were 40% and 20% at the 6‐month mark, respectively, while these rates increased to 53.3% and 40%, respectively, during the final follow‐up assessment (Malone et al. 2009). Bergfeld et al. (2016) demonstrated that ALIC‐DBS exhibited significant efficacy in reducing depressive symptoms in 10 out of 25 patients and was well‐tolerated; however, variations in electrode placement, optimization phase duration, and timing of DBS settings evaluation may yield divergent outcomes. The long‐term efficacy of ALIC‐DBS has also been reported, with a response rate of 44.4% and sustained efficacy observed during a 2‐year follow‐up period (van der Wal et al. 2020). Additionally, researchers have demonstrated that ALIC‐DBS does not impact the cognitive function of patients with TRD (Bergfeld et al. 2017). However, it is worth noting that certain adverse reactions, such as severe nausea and suicidal tendencies, have been reported during the surgical procedure (Bergfeld et al. 2016). However, a recent randomized controlled trial demonstrated that the response rates of ALIC‐DBS at 12 and 24 months were 20% and 23.3%, respectively, indicating no statistically significant difference compared to the control group (Dougherty et al. 2015). These short‐term null results may reflect limitations inherent to trial design, such as the use of sham‐controlled phases (e.g., 16 weeks) that are too brief to capture delayed antidepressant effects. Additionally, variability in lead placement accuracy and individual neuroanatomical differences could contribute to heterogeneity in trial outcomes.

### 3.5 Lateral Habenula

The LHb is situated on the dorsomedial surface of the thalamic tail, which has a triangular shape and extends towards the third ventricle. It mainly receives afferents from the thalamus, striatum, and limbic system and projects to the dorsal raphe nucleus, VTA, and substantia nigra, and indirectly regulates the hippocampus, hypothalamus, amygdala, and frontal cortex. The LHb is involved in a diverse range of functions, including reward processing, social interactions, behavioral adaptation, circadian rhythms, and sensory integration (Zhao et al. 2015; Baker et al. 2016). Meng et al. (2011) demonstrated that LHb‐DBS intervention significantly ameliorated depressive symptoms in rats, potentially attributed to elevated levels of monoamines (norepinephrine, dopamine, serotonin) in both serum and brain tissues. Sartorius et al. (2010) reported a case of depression that exhibited significant improvement after 4 months of LHb‐DBS; however, relapse occurred upon cessation of stimulation. Another case within the same research group demonstrated more than 50% improvement in TRD following treatment with LHb‐DBS (Kiening and Sartorius 2013), and an observed notable increase in peripheral blood brain‐derived neurotrophic factor (BDNF) levels was found among TRD patients (Hoyer et al. 2012). Due to the compact size of the LHb and its close proximity to the brainstem and third ventricle, targeting the afferent nerve pathway through electrical stimulation of the striatum emerges as a potentially safer approach (Kochanski et al. 2016).

### 3.6 Bed Nucleus of Stria Terminalis

The BNST is situated in the basal forebrain, adjacent to the ALIC and NAcc, and constitutes an integral component of the limbic system. It serves as a principal efferent structure of the amygdala, which plays a pivotal role in modulating stress responses that are implicated in the pathogenesis of depression (Lebow and Chen 2016). Currently, the research on BNST‐DBS treatment for TRD remains limited. In a case report, a patient with comorbid anorexia nervosa and TRD initially underwent MEB‐DBS but switched to BNST‐DBS after 2 years due to the side effect of blurred vision. Remarkable therapeutic efficacy was observed (Blomstedt et al. 2017). Fitzgerald et al. (2018) conducted BNST‐DBS treatment on five TRD patients, resulting in sustained remission for two patients, significant improvement for two patients, and only one patient exhibiting ineffectiveness to BNST‐DBS treatment. In another double‐blind trial, the researchers conducted a cross‐stimulation test of ITP and BNST in seven patients with TRD. The results demonstrated that BNST stimulation yielded superior outcomes compared to ITP stimulation at 16 months post‐operation. Subsequently, after three years of DBS implantation, all patients received BNST‐DBS treatment, resulting in sustained remission for two out of seven patients and symptom improvement for the remaining five individuals (Raymaekers et al. 2017). These experimental findings suggest that BNST‐DBS holds promising prospects for TRD treatment; however, further clinical trials are warranted to substantiate these claims.

### 3.7 Medial Forebrain Bundle

MFB is an important component of the dopamine reward circuit, which is a set of fibers connecting the cerebellum, basal forebrain (including the VTA and NAcc), hypothalamus, and projecting to the medial PFC (Coenen, Sajonz, et al. 2018). Previous studies have demonstrated that glutamate within the PFC exerts regulatory control over dopaminergic neuron activity in the VTA, thereby indirectly facilitating the activation of dopaminergic neurotransmission within this region. MFB‐DBS may modulate dopaminergic and glutamatergic neurotransmission, thereby enhancing neuronal activity in these regions, activating the midbrain‐cortical system, and exerting antidepressant effects (Fenoy et al. 2016), particularly in TRD patients with psychomotor retardation. Rapid symptom improvement in MFB‐DBS trials may reflect dopaminergic activation, a mechanism relevant to motor‐related depressive phenotypes.

Multiple studies have consistently reported that MFB‐DBS exhibits rapid and sustained antidepressant effects. The initial report by (Schlaepfer et al. 2008) introduced MFB‐DBS as a treatment for TRD, demonstrating that six out of seven TRD patients achieved a positive response within one week, with more than 50% improvement in depression scales. Following a 12–33 week observation period, all six patients exhibited sustained responses, with four of them meeting the criteria for complete remission (Schlaepfer et al. 2013). The results of the open trial conducted by (Fenoy et al. 2018) indicated that within one week, more than 50% of patients with TRD experienced a significant improvement in their depression scores. After 26 weeks, over 80% of patients showed an improvement of more than 80% in their depression scores (Fenoy et al. 2016). At the end of 52 weeks, more than 70% of patients demonstrated a depression score improvement exceeding 70% (Fenoy et al. 2018). The long‐term efficacy analysis conducted by (Bewernick et al. 2010) demonstrated that symptoms improved in six out of eight patients with TRD, and complete remission was achieved in four patients after 1 year, without any observed cognitive or personality changes (Bewernick et al. 2018; Bewernick et al. 2017). Notably, potential side effects such as blurred vision and strabismus were reported (Blomstedt et al. 2017; Bewernick et al. 2018). Additionally, Coenen et al. (2019) discovered that the enlargement of the left frontal pole and a portion of the orbitofrontal region can serve as predictive indicators for the therapeutic efficacy of MFB‐DBS, representing a crucial advancement toward personalized treatment.

### 3.8 Inferior Thalamic Peduncle (ITP)

The ITP establishes a bidirectional communication pathway between the dorsomedial thalamus and the orbitofrontal cortex, serving as a crucial link for information exchange. Previous clinical trials have demonstrated that electrical stimulation of the ITP effectively modulates the non‐specific thalamic‐orbitofrontal cortex circuitry, leading to notable antidepressant effects (Velasco et al. 2006). In addition to connecting the dorsolateral thalamus with the OFC, the fibers of ITP extend to the ALIC, the PFC, and the VS, integrating key components of the brain's emotional regulation framework (Kopell and Greenberg 2008). Jiménez et al. (2005) reported the results of DBS for ITP in two depression patients, both of whom exhibited the anticipated antidepressant efficacy without any adverse effects (Jiménez et al. 2013). Recently, a double‐blind crossover trial was conducted to compare the antidepressant efficacy of ITP and ALIC/BNST‐DBS. Despite no discernible advantage or disadvantage observed between the two targets, it is noteworthy that six of seven patients expressed a preference for ALIC/BNST‐DBS treatment (Raymaekers et al. 2017). Clearly, further exploration is warranted to fully understand the potential of ITP‐DBS for TRD.

### 3.9 Multi‐Targets

When searching for the optimal target for DBS, it is imperative to consider both the neural circuit heterogeneity of TRD and its clinical subtypes (e.g., anhedonia‐dominant vs. anxiety‐dominant phenotypes). Emerging evidence suggests target‐specific efficacy: SCG/NAcc for anhedonia, MFB for psychomotor retardation, and BNST for anxiety‐driven TRD. This underscores the need for phenotypic stratification in future trials. Moreover, there typically exists no singular ideal DBS target for depression treatment, as antidepressant effects can be achieved through the stimulation of various targets. Aberrations in the cortico–striato–thalamo–cortical loops have been proposed as a potential factor in the development of TRD (Mayberg 1997; Kopell et al. 2004). This neurocircuitry involved in depression is believed to encompass the dorsal (prefrontal, dorsal anterior cingulate, and premotor cortices), ventral (sACC, orbitofrontal, and insular cortices), and modulatory (pregenual ACC, amygdala, and the hypothalamic–pituitary axis) components (Kopell et al. 2004). For example, it has been hypothesized that applying DBS in an area where fibers from the ventral and dorsal compartments converge, such as the NAcc, may allow for simultaneous excitation and inhibition in the dorsal and ventral compartments, respectively (Drobisz and Damborská 2019, Kopell et al. 2004), thereby influencing the dysbalanced neural system in a complex manner.

## 4 Challenges and Perspectives

However, it must be acknowledged that the use of DBS for the treatment of TRD is still in the research stage. At present, several targets are being evaluated in clinical trials, with CG, VC/VS, and NAc showing more advanced progress, and slMFB also showing potential (Marwaha et al. 2023). However, the credibility of ITP, LHb, and BNST is limited due to the scarcity of studies and lack of repeated research support. Most clinical trials lack proper blind randomization and control, and the sample size is often relatively small. Additionally, there is a lack of strict control over the heterogeneity of participants' depression, leading to uneven treatment effects. A recent meta‐analysis revealed that the 1‐year response rate of different DBS targets in TRD ranged from 36% to 60% (Zhou et al. 2018). Among these, only ALIC and SCG were tested in multicenter, randomized, double‐blind controlled trials. However, no significant statistical differences were observed (Puigdemont et al. 2015, Holtzheimer et al. 2017, Dougherty et al. 2015), partially attributable to challenges unique to DBS trial design. For instance, short sham‐controlled phases (often ≤ 6 months) may inadequately capture the time‐dependent therapeutic effects of DBS, which require weeks to months for full manifestation. Furthermore, variability in lead placement accuracy (e.g., deviations from optimal fiber tract trajectories) and heterogeneous patient neurocircuitry likely contribute to inconsistent outcomes (Dandekar et al. 2018). Early DBS trials predominantly relied on anatomical coordinates for targeting, but recent advances emphasize tractography‐based approaches to map white matter pathways (e.g., MFB, ALIC) for precise electrode placement. This shift is critical, as stimulation efficacy depends on modulating specific fiber bundles rather than gray matter nuclei alone (Sadeghzadeh et al. 2024). Currently, there is no established stimulation parameter for the DBS treatment of TRD. Different stimulation parameters are primarily selected based on different target areas, highlighting the importance of personalized treatment.

Furthermore, DBS may elicit a series of adverse reactions. Multiple studies have documented instances of suicide and loss of follow‐up during DBS treatment, suggesting that stimulation may exacerbate cognitive impairment (Narang et al. 2016; McInerney et al. 2017). An assessment was conducted to evaluate the impact of SCG‐DBS on cognitive ability (McInerney et al. 2017). It was found that TRD patients performed worse compared to the healthy control group, but SCG‐DBS did not show any deterioration in neuropsychological function. Additionally, post‐processing speed and executive function showed improvement at 6 months, and cognitive function did not deteriorate at 1 year. It is suggested that suicide motivation may have originated from other life events. On the contrary, SCG is associated with the neural network of dopamine‐related psychomotor processing, including the ventral striatum, NAcc, amygdala, and PFC. Treatment with SCG‐DBS can improve the severity of psychomotor delay in patients, suggesting that SCG‐DBS may have a promoting effect on dopamine function. Previous studies have found no evidence of cognitive decline during IC/BST or ITP stimulation (Raymaekers et al. 2017). The improvement of depression symptoms in patients is dependent on the enhancement of neurocognition. However, it cannot be concluded at this time that cognitive impairment suicide is not related to DBS treatment. In addition, several studies have indicated that acute stimulation during VC/VS, NAcc, and MFB implantation testing may result in adverse reactions such as mild mania, anxiety, tension, and sensory abnormalities (Schlaepfer et al. 2014). Mild mania is the most frequently reported reaction (Narang et al. 2016), with others including transient confusion of consciousness, emotional changes, sleep disorders, memory disorders, appetite changes, weight gain, and increased libido (Zhou et al. 2018). These findings have contributed to skepticism among psychiatrists and patients regarding the use of invasive DBS treatment for TRD, which is not conducive to the conduct of clinical trials or the recruitment of trial subjects.

In recent years, personalized guidance of DBS targets and parameter selection is anticipated to further enhance clinical treatment effectiveness.

Currently, research has commenced to identify the biological markers of individuals in a depressive state based on the neural network characteristics of patients with TRD, such as closed‐loop therapy, which is a novel DBS method. Unlike traditional DBS treatment, which provides continuous stimulation, closed‐loop therapy aims to deliver short, intermittent stimulation when patients are in the target state according to their biological markers in a depressive state (Scangos KW, Makhoul GS, et al. 2021). Potential biomarker signals, including LFPs and intracranial electroencephalography (iEEG) signatures, may be used to trigger or modulate stimulation in real‐time. However, these biomarkers for depressive states can differ significantly across individuals due to neurocircuit heterogeneity (Sadeghzadeh et al. 2024). Therefore, truly personalized, adaptive DBS requires patient‐specific mapping of neural activity patterns rather than relying on a “one‐size‐fits‐all” biomarker. Scangos KW, Makhoul GS, et al. (2021) proposed the implantation of multi‐site electrodes in the brain to identify areas related to emotional stimulation. This approach aims to utilize individualized neural data to determine stimulation targets and strategies, effectively alleviating depressive symptoms. The team utilized closed‐loop therapy to stimulate the right VC/VS target in a patient with TRD. The study initially involved implanting intracranial multi‐site electrodes to identify the EEG signature associated with depressive symptoms and then administering DBS when the EEG signature was present. As a result, the patient's depressive symptoms showed rapid and sustained improvement (Scangos KW, Makhoul GS, et al. 2021). This new concept provides a novel and promising treatment approach for future TRD treatment.

## 5 Conclusions

In conclusion, DBS is still considered an experimental therapy in TRD. Although the mechanism, optimal brain structure, and appropriate biomarkers of TRD‐DBS have not yet been determined, the advantages of brain structure electrical stimulation compared to other treatment programs are evident. It is completely reversible and can be adjusted to accommodate differences in demand between individuals as well as within the same individual, which may arise due to the presence of different disease subtypes and disease progression. This provides a new treatment option for patients with TRD who have not responded well to traditional therapies. In the future, personalized stimulation targets can be determined based on the clinical characteristics of each patient and combined with biological markers, such as stereotactic electroencephalography, direct intracranial stimulation feedback, and neurophysiological recording. Brain imaging methods can be utilized to accurately guide the implantation of stimulation electrodes into the brain target, as well as to optimize DBS devices and treatment strategies for different target locations, such as unilateral or bilateral stimulation, pulse width, frequency, closed‐loop stimulation decoding biomarkers, and so forth. This personalized and precise DBS treatment can further improve the effectiveness of TRD treatment, reduce adverse reactions, and improve patient tolerance.

## Author Contributions

**Jianyang Dong**: writing – review and editing. **Mengying Dai**: writing – original draft. **Zinan Guo**: writing – review and editing, software. **Ting Xu**: resources. **Fangming Li**: investigation, formal analysis. **Jianjun Li**: conceptualization, supervision.

## Ethics Statement

The authors have nothing to report.

## Consent

The authors have nothing to report.

## Conflicts of Interest

The authors declare no conflicts of interest.

### Peer Review

The peer review history for this article is available at https://publons.com/publon/10.1002/brb3.70505

## Data Availability

The datasets generated and/or analyzed during the current study are available from the corresponding author upon reasonable request.
